# The Foreign Journals: Pathology, Practical Medicine, and Therapeutics

**Published:** 1841-07

**Authors:** 


### PATHOLOGY, PRACTICAL MEDICINE, AND THERAPEUTICS.

### *On Croup and its Cure.* By Dr. Grahl of Hamburgh.

Dr. Grahl calls attention to the employment of arm-baths in croup, the use of which has already been advocated by Dr. Dorfmüller in the fifty-first volume of Rust's Magazine. They are indicated in the commencement of the stage of exudation, the existence of which is rendered evident by the difficult respiration. He regards the employment of leeches in this stage as of no avail, and the administration of sulphate of copper and emetics as likely to increase the exudation. His recommendation is that the arms of the patient be placed in a vessel sufficiently deep to admit them to a hand's breadth above the elbow-joint and filled with water as hot as it can be borne. A cloth should now be thrown over the head of the patient, which, falling down around the edges of the bath retains the vapour, and this the patient should be allowed to respire for a quarter of an hour together, repeating it at short intervals. The first application usually induces some degree of moisture in the Schneiderian membrane, and diminishes the dyspnœa. As it is repeated the cough usually loses its hoarse tone and the patient expectorates the exuded lymph. In cases where the symptoms are extremely urgent, calomel in large doses should be given and a blister applied to the throat, but in the great majority of cases the employment of the arm-bath is all that is necessary.

*Zeitschrift für die gesammte Medicin. Band* xvi. *Heft* iv. S. 496.

---

### *Communication of an Intermittent Fever from a Mother to her Child.* By Dr. Brunzlow, of Brandenburg.

The mother suffered throughout nearly the whole of the last seven months of her pregnancy with her first child from tertian and then from quartan ague. The child was born very weakly, and after some weeks of more or less illness the mother noticed that it appeared much worse on some days than on others. On closely watching it, the author found that every fourth evening the infant became very cold and had its nails quite blue, and that then a considerable degree of heat came over the whole body, and was followed by slight sweating. On the next morning all these symptoms had disappeared, and for the next two days the child remained perfectly well. The evidence of its being actually affected with quartan ague was further strengthened by its being slowly cured by the administration of quinine. *Medicinische Zeitung. Marz* 24, 1841.

*Poisoning of Leeches by the Blood of the Sick.* By Stefano Grandoni.

In a paper containing experiments to prove that portions removed from leeches are not reproduced, the author relates the following interesting case:

In the beginning of May there was brought to the Brescia Hospital for Males a butcher, who had contracted malignant carbuncle in cutting up an ox that had died of that disease. Among the means employed were 120 leeches, which were to be applied in three divisions to his right arm, all of which died on being taken off the skin of the patient. Our chemist being informed of the case, though he did not doubt that the death of the leeches proceeded from the blood which they had sucked and were full of, had several other lively and strong leeches applied, and these also immediately fell off dead. This having occurred in the calamitous time of the cholera, curiosity was excited to know whether leeches would also die from the effects of the blood sucked from cholera patients, and it was found on good information that this had actually occurred in the cholera hospital.

*Commentarij dell' Ateneo di Brescia,* 1837-8.
*Annali Universali di Medicina, Ottobre,* 1839.

---

*On the Treatment of Acute Articular Rheumatism by large doses of the Nitrate of Potash.* By M. Aran.

This is a very long paper, detailing the results of twelve cases of acute rheumatism treated by MM. Gendrin and Martin Solon. Of these, ten were males, two females, from the ages of sixteen to forty; the stage of the disease varying from the second to the fifth day, and the previous treatment having been unimportant. The nitrate of potash was administered in some demulcent ptisan, the mean quantity taken by each patient being *an ounce and two scruples per diem,* and twelve ounces throughout the whole duration of the disease. The mean duration of the disease was eight days from the commencement of the treatment and rather more than twelve from the period of invasion. The usual effects were very abundant perspirations; occasionally copious stools, but without colic; and free secretion of urine. The digestive organs were unimpaired in their functions; only one patient vomited; the pulse was depressed; and the pain in the joints rapidly diminished. In three cases complicated by endocarditis or pericarditis, these affections rapidly subsided, though no other remedies were employed. Only two patients suffered a relapse, and they were again cured by the same means.

[This treatment is not new, for Brocklesby and White in this country, towards the close of the last century, gave nitre from ten drachms to two ounces in the twenty-four hours, and spoke highly of its efficacy both in acute and chronic rheumatism. However the mean duration of the disease under its use does not appear to be less than is obtained by other means. Dr. Hope, who trusted to calomel and opium, states the usual duration to be eight or ten days, and we have frequently seen severe cases rapidly cured by colchicum and opium. Bouillaud, who employs free bleeding, gives one or two weeks. Dance, who gave the tartarized antimony freely, forty to sixty days. The nitre is evidently preferable to the two latter.]

*Journal des Connaissances Médico-Chirurgicales. Avril,* 1841.

---

*On the Pathology of Plica Polonica.* By Dr. Bidder, of Dorpat.

In a paper on the structure and physiology of the human hair, the author has communicated the following interesting observations on this disease, which he had an opportunity of making, during a residence of several weeks in a district in which it was very prevalent, though not in its severest form.

In none of the cases examined did the matting of the hair extend to the scalp, but for a distance of between half an inch and an inch from it all the hairs, however matted in the rest of their length, were perfectly normal. It might be imagined indeed, that healthy hair had in these cases begun to grow again, and that in an earlier stage of the disease, the whole had been matted together; but the same appearance having been observed in at least twenty individuals placed under a variety of different circumstances, proves that it is a constant and essential fact. Moreover, since the scalp beneath the matted hair was perfectly healthy, exhibiting neither redness nor swelling, nor increased sensibility, it is impossible to refer the morbid appearances in the hair to disease of its matrix. It must rather be held, that in consequence of a morbid process commencing in a defined part of their constituent fibres, the hairs stick together in bundles of various sizes, and become individually so loosened in their texture, that they split up into smaller and finer portions. The frequent occurrence of such fine hairs, as well as that of the much coarser hair in the plica, can scarcely be otherwise explained.

A fact still more remarkable, and affording a still stronger proof of the vitality of the hair, is a process of separating or shedding which occurs in some cases of plica. The author saw two persons from whom plicæ had lately fallen off spontaneously, without leaving any part of the head bald; and indeed this occurrence is regarded by the people of the district, as a favorable though a rare symptom in the disease. In two others he was able to trace the mode in which the separation takes place. In these, there formed at the limit between the diseased and healthy hair, in the tuft that was matted beyond a certain distance from the scalp, a deep groove which completely encircled the tuft as if a string had been tied round it. It formed a very distinct boundary between the two parts of the tuft, and as it went on, the constriction becoming gradually deeper, a part of the hairs separated, and the whole plica was held by only the remainder of those by which it was originally constituted, and at last it entirely fell off. The whole process bore a remarkable resemblance to that of the casting off of other diseased parts, such as occurs, for example, in gangrene; and the whole series of the phenomena are explicable only on the belief that they depend on an action occurring in the hair itself.

*Müller's Archiv.* 1840. *p.* 546.

---

*On Horny Excrescences on the Eyelids.* By Dr. F. A. v. Ammon.

Professor Stromeyer, of Erlangen, had the kindness to send me a drawing of a horny growth, with the following description: "This horn has existed ten weeks, and still goes on growing; six weeks since the excrescences on the nose formed, and these are now ready to fall off. The horny mass does not proceed from a follicle, but was probably secreted by the vascular skin at its base. It gives no annoyance; the closing of both the eyelids is effected only imperfectly from want of tone, and the conjunctiva is somewhat loose. The patient is a woman, seventy-one years old." Thus far Stromeyer.

To this interesting communication I may add the following. I have twice observed horny growths on the eyelids. The cases were as follows: A stocking-maker, forty years old, healthy and strong, had a horny growth, three lines large, on the right upper eyelid, a few lines from its ciliary margin. It had grown gradually, and turned downwards. The patient believed that it came from a wart; and he came to me only because he had lately often struck the growth, and given himself pain. I cut it off with scissors, going rather deep into the healthy skin, and the wound bled considerably for some time. When it had ceased bleeding I touched it with nitrate of silver, and in twelve days it was completely healed. Two years have elapsed, and the disease has not re-appeared.

The most accurate examination detected nothing more than that the horn was a dark cartilage-like substance, uniform from its base to its apex, and consisting of several close lamellæ. Where it was connected with the skin, there was seen an intermediate substance like a thickened cutaneous gland.

In the second case, I observed the disease in a woman fifty years old, who had ceased menstruating for some time, and had had warts on many parts of her body successively, which, however, were always removed by the remedies she applied. From a wart on the left upper eyelid, a horny excrescence had gradually formed, which was upwards of four lines high, inclined to one side, and smaller at its apex than at its base. It gave no pain, but it often itched, and when the woman scratched it, it bled. I extirpated it with scissors, and it has not been reproduced.

As far as I know, among the cases of horny excrescences described by authors, there is none of a *cornu cutaneum* of the eyelids.

*F. A. v. Ammon's Monatsschrift für Medicin n. a. Juli,* 1840.

---

*Poisoning by Solution of Acetate of Lead.—Lead found in the Urine.*

A young girl of good constitution, driven by despair to suicide, took about an ounce of acetate of lead in solution. She was almost immediately seized with collapse and syncope, and afterwards with vomiting and convulsions. Sugared water, sulphate of magnesia, and sulphate of soda were given, but she died in twenty-five hours. She voided a large quantity of urine, which M. Villeneuve sent to M. Orfila. Carbonized, treated by nitric acid, and submitted to the tests of the salts of lead, this urine afforded a sensible quantity of lead. *Journal des Connaissances Médico-Chirurgicales. Janvier,* 1841.

---

*Antimony in the Urine.*

AT the sitting of the Royal Academy of Medicine, Dec. 8, 1840, M. Husson stated that he had given a scruple of tartar emetic to a patient affected with pneumonia in twenty-four hours. It produced neither stools nor vomiting, and on the urine being examined by M. Orfila, with Marsh's apparatus, it afforded the antimonial stains in great abundance.

*Archives Générales de Médecine. Janvier,* 1841.

---

*On the Employment of Arsenious Acid in Phthisis Pulmonalis.* By M. TROUSSEAU.

M. TROUSSEAU is now making trial of the powers of arsenic in phthisis at the hospital Necker; a means, however, not new, as Dioscorides employed fumigations of the sulphuret of arsenic in the same disease. During some few months M. Trousseau has submitted eight patients to the action of this agent. In four affected with diarrhœa, the disease continued its progress; it was in an advanced stage, and death occurred in the usual manner. In four others, notwithstanding vast caverns, and symptoms which announced approaching death, the symptoms amended under the influence of arsenious fumigations, the general health improved, appetite returned, digestion was good, emaciation checked, and the cough and expectoration diminished.

A sheet of white paper is dipped in a solution composed of one part of arseniate of soda and thirty parts of water. The paper is then made into little cigars of the length of a finger, and the patient is directed to smoke one or even two daily, in such a manner that the fumes may pass into the lung. This is readily accomplished, by the patient inspiring the moment the fumes enter the mouth. This inspiration of arsenious vapour at first causes slight cough, but after some time both cough and expectoration are much diminished.

This means, though it has not as yet cured phthisis, deserves a trial. In one case of chronic catarrh with emphysema, it rapidly removed symptoms of suffocation. Careful experiments should be made with this remedy, for phthisis is a disease hitherto so incurable that anything which affords any probability of effecting permanent good is worthy of investigation.

*Bulletin Général de Thérapeutique. Février* 15 *et* 28, 1841.

*On Scorbutic Pericarditis and Pleuritis, and the Cure by Paracentesis.* By Dr. Karawagen, of Kronstadt.

The scurvy epidemic which prevailed among the sailors at Kronstadt during the months of May, June, and July in 1839, was particularly marked by the effusions of blood into the cavities of the chest which are known by the names of *pleuritis* and *pericarditis scorbutica*. The common sign of both these effusions, which commonly take place very rapidly, is an extreme difficulty of respiration. In the pericarditis the heart's sounds appear faint and remote, and percussion yields a dull sound over an unnaturally wide extent. In the pleuritis, only a weak respiratory murmur can be heard on the affected side; percussion is dull, and the respiration is short and hurried. There are also present slight fever and most of the ordinary signs of severe scurvy, which indeed always precede by one or more days the more dangerous symptoms of the thoracic effusions. When the latter come on, the condition of the patient is at once one of imminent danger, and the pericardial hemorrhage is rarely survived beyond the third day.

In the examinations of sixty patients who died of scurvy, the author found that thirty had this kind of pericarditis, twenty-two had pleuritis, two pleuritis and pericarditis together, and two peritonitis. In the pericarditis the pericardium appears three times as large as natural. It contains a bloody, dark-red exudation, which may amount to as much as four or five pints; so that it might be suspected that some vessel had been ruptured, though on examination none such can be found. The heart itself appears three times as small as usual; its surface, as well as the lining of the pericardium, is covered with a layer of red mould-like substance that may be scraped off with a knife; and beneath this the substance of the heart is soft and fatty. The endocardium is red, probably from imbibition; the ventricles are greatly compressed, and always empty; the left lung is compressed and bloodless. When there has been pleuritis, ten or twelve pints of bloody fluid are found in the pleuræ, whose surface is similarly covered by a layer of mould-like substance, and is very red and vascular. In peritonitis the appearances are similar, but the quantity of fluid amounted (in the cases examined) to from thirty to thirty-five pints. Once, in a case in which the patient died with symptoms of apoplexy, a similar effusion was found in the cavity of the skull. Sometimes the bones and fibrous tissues were affected with scurvy, and this was especially the case with the ribs, whose ends were found separated from their cartilages, moving on them with a kind of crepitation, and looking as if they had been eaten away by caries.

In despair of any other remedy, the author determined on tapping the serous cavities into which the hemorrhage had taken place. He performed this operation six times: on four patients with pleuritis, and on two with pericarditis; one of the last recovered completely, and all were immediately relieved and had their lives prolonged. The case of the man whose life was saved, and who was the only patient that recovered after pericardial effusion had evidently taken place, is related at length. He appears to have been in extreme peril when the operation was performed; but directly after three pints and a half of fluid had been extracted he felt much relieved, though a quantity of air had passed into the pericardium through the trocar; and he gradually recovered under the use of the ordinary antiscorbutic and some other means. Five months after the operation he might be considered quite well.

*Medicinische Zeitung. December* 23, 1840.

---

*On certain Anthelmintics in use in Abyssinia.* By Dr. Aubert.

Dr. Aubert, who has resided some time in Abyssinia, states that the whole medical practice of the natives consists in the employment of three indigenous vegetable anthelmintics called Cusso, Bisenna, and Abbatsjogo; and that tænia is so common among the inhabitants as to be considered almost natural. Bruce remarked that almost all Abyssinians were affected with ascarides; but as the

Abyssinian word applies to either species, and Bruce was not a physician, it is probable he confounded the two, especially as the cusso, which he praised as a remedy against these worms, Dr. Aubert finds used in tænia.

The cusso is the Banksia Abyssinica of Bruce, the Hagenia anthelmintica of Willdenow, one of the species of the genus Saponaria of Linnæus. M. Kunth, an able botanist, has recognized the flowers as a new genus of the Rosaceæ. About two drachms of the flowers in powder are taken in an electuary of honey, and ordinarily in about twenty-four hours the patient passes the worm, but without purging. The whole of the worm is rarely expelled; but the patient follows his usual occupation, and after a time, as the medicine is not unpleasant and does not cause griping or purging, the dose is repeated. Dr. Aubert became a subject of tænia whilst in Abyssinia, and by taking aperient medicine after the cusso, he completely relieved himself of it.

The bisenna is a species of conifera much resembling the Juniperus Virginiana of Linnæus. The pulverized bark is employed, but not so generally as the cusso, as it causes irritation, colic, and in some cases enteritis.

The abbatsjogo resembles the Ixia bulbocodium of Linnæus. It is less employed than either of the former species.

*Bulletin de l'Académie Royale de Medécine. Mars* 15, 1841.

